# Coronary Flow Assessment in Unstable Angina/non ST-segment Elevation Myocardial Infarction Patients via Thrombolysis in Myocardial Infarction Frame Count in Angiography

**DOI:** 10.5812/cardiovascmed.9087

**Published:** 2013-05-20

**Authors:** Hamidreza Sanati, Ali Zahedmehr, Ata Firouzi, Negar Salehi, Mohsen Maadani, Farshad Shakerian, Reza Kiani, Pedram Golnari, Sepideh Parchami-Ghazaee, Mohammadmehdi Peighambari

**Affiliations:** 1 Cardiovascular Intervention Research Center, Rajaie Cardiovascular Medical and Research Center, Tehran University of Medical Sciences, Tehran, IR Iran

**Keywords:** Angina, Unstable, Myocardial Infarction, Coronary Angiography

## Abstract

**Background:**

TIMI Frame Count (TFC) is one of the methods to estimate the coronary blood flow velocity. This is a simple, inexpensive, quantitative, reproducible, and continuous variable method. Many studies have been conducted on TFC assessment in ST elevation myocardial infarction (STEMI) patients.

**Objectives:**

The present study is aimed to measure the TFC in the coronary arteries of UA/NSTEMI patients to find abnormalities in diseased or patent vessels and compare with the normal values.

**Patients and Methods:**

The participants were 105 consecutive UA/NSTEMI patients who underwent coronary angiography in Shahid Rajaie Cardiovascular Medical and Research Center, Tehran, Iran in 2009. Exclusion criteria were history of CABG, PCI, or STEMI or presence of occluded arteries in angiography. We measured the coronary TFC in these patients. We examined also 55 stable patients without coronary lesions and with TIMI 3 flow to have an estimation of normal TFCs.

**Results:**

From a total of 105 patients, 25 (23.8%) had no significant coronary lesion (> 60%); 35 (33.3%) were diagnosed with single vessel disease; 22 (21%) were 2VD; and 23 (21.9%) were 3 VD.). In overall, mean TFC in UA/NSTEMI group was 28.7 (± 14) frames compared to 23.8 (± 7.8) frames in the normal group (P < 0.05). In the vessels with significant lesions, TFC was significantly higher than normal (30.84 vs. 23.8; P < 0.001) and also significantly higher than patent vessels of the same patients (30.84 vs. 26.10; P = 0.029). In these patients, patent vessels had higher TFC values compared to normal coronaries (26.10 vs. 23.8), but the difference was not significant (P = 0.12). In the patients with significant lesions, mean TFC was higher than the same value in acute coronary patients without significant lesions (29.3 vs. 27.2), but the difference was not significant (P = 0.114). In the patients who underwent PCI and stenting, TFC changed significantly after PCI toward the normal value (P = 0.001). In the patients with elevated cardiac enzymes, TFC was higher but the difference was not significant (P = 0.35).

**Conclusions:**

Patent coronaries of UA/NSTEMI patients have a trend to higher TFCs compared to normal values. Presence of significant coronary lesions in these patients significantly increases TFC.

## 1. Background

Atherosclerosis is an important cause of morbidity and mortality all around the world. Coronary angiography is the procedure of choice for evaluation of severity and burden of disease in epicardial portions of coronary arteries. Blood flow velocity is a parameter which is influenced by both epicardial and microvascular portions of coronaries (1). Accordingly, it can give information about invisible parts of coronary arteries. TIMI Frame Count (TFC) is one of the methods to estimate the coronary blood flow velocity. This is a simple, inexpensive, quantitative, reproducible, and continuous variable method. The number of frames needed until the contrast media opacify the total length of a vessel from the beginning to the standard distal landmarks is counted (2). The correlation of this method with the results of doppler has been demonstrated (3). The well-known TIMI flow assessment, which describes coronary flow as categories of 0 to 3, has some drawbacks. The most important is interobserver variability. TFC is more accurate in this regard. Many studies have been conducted on TFC assessment in ST-segment elevation myocardial infarction (STEMI) patients (2, 4-6). TFC is abnormal in nonculprit vessels in acute MI setting which shows improvements after 18-36 hours (2). Nonculprit vessels in UA/NSTEMI patients may have the same condition.

## 2. Objectives

Our aim was measuring TFC in UA/NSTEMI patients' vessels and we wanted to know whether coronary blood flow velocity was reduced in patent vessels as well as vessels with significant lesions.

## 3. Patients and Methods

This study was conducted on 105 UA/NSTEMI patients who underwent coronary angiography in an initial invasive strategy. Patients with a history of coronary artery bypass grafting surgery, percutaneous coronary intervention (PCI), STEMI, or occluded arteries on angiography were excluded from the study. Additionally, 55 stable patients with angiographically normal coronary arteries and TIMI 3 flow were examined to obtain an estimation of normal TFC values. Demographic data and information about the cardiac risk factors, Left Ventricular Ejection Fraction (LVEF), biochemical markers, and medications were extracted from medical records. Angiography was performed via femoral access by 6F catheters in all patients without nitroglycerine administration. 

A single observer measured the TFC as frames which last to dye opacification of the entire length of a coronary vessel from the beginning where the hand injected dye touches both sides of a coronary ostium and has an antegrade flow to the standard distal land marks. For the left anterior descending artery (LAD), the distal bifurcation (i.e., the mustache, pitchfork, or whales tail); For the left circumflex artery (LCX), the distal bifurcation of the segment with the longest total distance that includes the culprit lesion, and for the right coronary artery (RCA), the first branch of the posterolateral artery were considered as distal landmarks (2). We used angiography systems with digital acquisition speed of 12.5 or 15 frame/sec. To measure the TFC, the standard speed is 30 frame/sec; therefore, we multiplied the resulted values by 2 in 15 frame/sec systems and by 2.4 in 12.5 frame/sec systems. Regarding longer length of the LAD, TFC should be divided to 1.7 to adjust its value to LCX and RCA. This value is called CTFC (corrected TFC) (2). TFC adjustment was not performed for the heart rate. 

Data were represented as frequency (%) or mean (SD = standard deviation). Student t test was used when comparing TFCs (as a continuous variable) between two groups and one-way analysis of variances was applied when comparing more than two groups. A P value of < 0.05 was considered statistically significant, with all P values being two-tailed. Analyses were conducted with SPSS version 17 for windows (SPSS Inc, Chicago, Illinois, USA).

## 4. Results

Among 105 patients under study, 73 (69.5%) were men. Mean age was 56.4 (± 10.3) with a range of 36 to 81 years. Other baseline characteristics are outlined in Table 1. Of these patients, 25 (23.8%) had no significant coronary lesion (> 60% diameter stenosis); 35 (33.3%) had single vessel disease (SVD); 22 (21%) were 2VD; and 23 (21.9%) were 3VD.

**Table 1.** Baseline Characteristics of Study Patients

Characteristics ^a^	UA/NSTEMI patients	Normal coronary group
**Male**	73 (69.5)	15 (27.3)
**LV EF^b^> 50**	49 (46.7)	37 (62.3)
**Diabetes**	27 (25.7)	8 (14.6)
**Smoking**	25 (23.8)	10(18.2)
**Hypertension**	32 (30.5)	19 (34.6)
**Dyslipidemia**	47 (44.8)	20 (36.4)
**Family history**	13 (12.4)	4 (7.3)
**Stabilized**	82 (78.1)	-
**Chest pain in cath lab**	9 (8.6)	-
**Raised CKMB^b^**	69 (65.7)	-
**Raised Troponin**	63 (60)	-
**Preloaded Plavix**	91 (86.7)	-
**LAD^b^lesion**	62 (59)	-
**LCX^b^lesion**	47 (44.8)	-
**RCA^b^lesion**	39 (37.1)	-

^a^ Values are n (%)

^b^ Abbreviations: LV EF: left ventricular ejection fraction; CPKMB: creatinin kinase-MB; LAD: left anterior descending artery; LCX; left circumflex artery; RCA: right coronary artery

Mean LAD CTFC was 28.26(± 12.8) frames; Mean LCX TFC was 31.59 (± 16.8) frames; and mean RCA TFC was 26.2 (± 14.2) frames (Table 2). On the other hand, in 55 stable patients with normal coronary arteries, mean CTFC was 23.2 (± 6.6) frames for LAD, 25.3 (± 7.7) frames for LCX, and 22.9 (± 8.9) frames for RCA. In general, mean TFC in UA/NSTEMI group was 28.7 (± 14) frames compared with 23.8 (± 7.8) frames in normal group (P = 0.004).

**Table 2.** Coronary TFC in UA/NSTEMIPatients

Name of Vessel	TIMI Frame Count ^a^		P value
	With Lesion	Without Lesion	
**LAD^b^**	30.06 ± 14.39	25.65 ± 9.87	0.065
**LCX^b^**	33.12 ± 20.12	30.34 ± 13.56	0.420
**RCA^b^**	30.10 ± 18.69	23.95 ± 10.46	0.068
**Total**	30.84 ± 17.58	26.64 ± 11.87	0.029

^a^ values are mean ± SD

^b^ Abbreviations: LAD: left anterior descending artery; LCX: left circumflex artery; RCA: right coronary artery

Totally we had four vessel groups:

1. Vessels with significant lesions (n = 148; mean TFC = 30.84)
2. Patent vessels in the patients with significant lesions (n = 92; mean TFC = 26.10)
3. Vessels in the unstable patients without significant lesions (n = 75; mean TFC = 27.22)
4. Vessels in the normal group (n=165; mean TFC = 23.8)

In the vessels with significant lesions (group 1), TFC was significantly higher than normal (group 4) (30.84 vs. 23.8; P < 0.001) and also significantly higher than patent vessels of the same patients (group 2) (30.84 vs. 26.10; P = 0.029). Patent vessels in these patients had higher TFC values than normal coronaries (26.10 vs. 23.8), but the difference was not significant (P = 0.12)

In the patients with significant lesions (n = 80), mean TFC was higher than the same value in acute coronary patients without significant lesions (29.3 vs. 27.2), but the difference was not significant (P = 0.114).IIn the number of the patients who underwent PCI and stenting, TFC after PCI changed significantly toward normal (P = 0.001).

In the patients with elevated cardiac enzymes, TFC was higher but the difference was not significant (P = 0.35).

TFC was not correlated with clinical stability of the patient (being free of chest pain for a few hours). Also, the relation between TFC and LVEF, different risk factors, age, sex, height, weight, and intake of preloaded Plavix was not significant.

## 5. Discussion

TIMI flow is currently the method of choice for the evaluation of the coronary flow on a cineangiogram. Be that as it may, this favorable method is not entirely devoid of pitfalls, first and foremost amongst which are its significant interobserver variability and its diagnosis of many patients with different blood flow velocities as normal (TIMI 3 flow) (3). TFC is a quantitative, reproducible, and objective index of the coronary flow that allows standardization of TIMI flow grades (2). Previous studies have shown that in STEMI patients, TFC in nonculprit vessels is transiently impaired but gradually tends to normalize after stabilization (18-36 hours). Moreover, in the occluded artery, TFC after primary PCI is deemed as a predictor of mortality and future restenosis (2, 5, 6). 

The aim of this study was to assess TFC in UA/NSTEMI patients and to find any possible abnormalities in diseased or seemingly patent arteries. In our 55 individuals with normal coronaries, the mean CTFC was 23.8. In one of the previous studies, normal TFC was determined to be 21.3 (2) and 23 in another one(5). According to our results, vessels with significant lesions had TFC values, which were significantly higher than normal and also significantly higher than the values of with the values of patent vessels in the same patients. It seems that in patients with significant lesions, patent vessels have somehow abnormal TFC values: this could be assumed as microvascular disease. Our ACS patients without significant coronary lesions had a similar condition; consequently, we could optimistically look at TFC as a tool to differentiate between cardiac and non-cardiac chest pains in patients with patent coronaries in angiography. 

In a broad spectrum of patients with an abnormal coronary blood flow, TFC can be an index of the whole coronary vasculature, including epicardial parts and microvasculature; and therefore, acts as a "Coronary Flow Reserve" (CFR) index.

Previous studies have shown a relationship between TFC and CFR (7). Higher TFC values are signs of more severe vessel involvement and the culprit lesion can be expected to have the most abnormal TFC. Nevertheless, we had no definite criteria to determine the culprit lesion in our UA/NSTEMI patients. It may be possible to place the TFC data to information from the electrocardiogram, angiographic appearance of lesions, and presence of thrombi in order to identify the culprit lesion.
